# Willingness and Influencing Factors to Receive COVID-19 Vaccination Among Chinese Medical Students

**DOI:** 10.3389/fpubh.2022.869838

**Published:** 2022-06-03

**Authors:** Huan Liu, Zhiqing Zhou, Xiubin Tao, Long Huang, Ergang Zhu, Liang Yu, Shaoling Du, Ming Zhang

**Affiliations:** ^1^Department of Hemodialysis, The First Affiliated Hospital of Wannan Medical College (Yijishan Hospital of Wannan Medical College), Wuhu, China; ^2^Department of Nursing, The First Affiliated Hospital of Wannan Medical College (Yijishan Hospital of Wannan Medical College), Wuhu, China; ^3^School of Humanities and Management, Wannan Medical College, Wuhu, China; ^4^School of Comprehensive Foundation, Wannan Medical College, Wuhu, China; ^5^School of Innovation and Entrepreneurship, Wannan Medical College, Wuhu, China

**Keywords:** willingness, influencing factors, COVID-19 vaccine, Chinese, medical students

## Abstract

**Objectives:**

The aim of this study is to evaluate the desire of medical students in China to get vaccinated or not get vaccinated and the reasons for either decision.

**Methods:**

A cross-sectional survey was conducted from 11 March and 12 March 2021, by administering an online questionnaire to the Chinese medical students. Data entry and analysis were conducted using IBM SPSS ver. 26.0.

**Results:**

Of 3,047 students who completed the survey, 37.9% (1,154) of participants indicated that they would be vaccinated against COVID-19, while 62.1% (1,893) declared that they would not. Attitudes to the COVID-19 vaccine (*p* = 0.000), levels of eHealth Literacy (*p* = 0.000), the impact of COVID19 (*p* = 0.000), concerns about the COVID-19 vaccine (*p* = 0.000) and gender (*p* = 0.000) strong associations with willingness to receive the COVID-19 vaccine.

**Conclusion:**

The willingness to receive COVID-19 vaccination was sub-optimal among medical students in China. Educational interventions to improve medical students' perceptions and acceptance toward the COVID-19 vaccine are needed.

## Introduction

The coronavirus disease 2019 (COVID-19) pandemic has spread rapidly worldwide, and caused an unprecedented global disease burden. By 30 July 2021, there have been 196,553,009 confirmed cases of COVID-19, including 4,200,412 deaths (1, 2). The way to stop the spread of COVID-19 is to adopt strict epidemic response measures, including national lockdown and preventive measures, such as social distancing and mask-wearing (3–5). Due to the socio-economic burden associated with COVID-19, and as vaccination is one of the most cost-efficient and successful health interventions to prevent infectious diseases, a vaccine against COVID-19 maybe the best hope to end this burden (6).

Mathematic modeling suggested that 75% coverage is needed to reach the herd-immunity threshold to extinguish the ongoing pandemic. Vaccination for medical students is an important part of achieving herd immunity of Chinese citizens (7). Therefore, strategies to improve COVID-19 vaccinations coverage are essential to curb the COVID-19 pandemic. The Chinese government announced the implementation of the free COVID-19 vaccination program on January 9, 021. Understanding the data on Chinese medical students' intentions for the COVID-19 vaccines will help to formulate immunization policies and improve the vaccination rate in China.

Medical students are viewed as an insightful group of young people, so special attention needs to be paid to assessing their attitude toward COVID-19 vaccination. It is possible that medical students think they would not likely to take care of COVID-19 positive patients, and the infection control procedures are sufficient to protect them from acquiring the virus. Furthermore, medical students felt that younger, healthy people like themselves are at lower risk of acquiring severe COVID-19 infection, this might have affected their perceptions on COVID-19 vaccination. Therefore, it is very important to study the COVID-19 vaccination acceptance among medical students in the context of the COVID-19 global pandemic. To our best knowledge, COVID-19 vaccination acceptance and associated factors among medical students in China has not been studied since the first COVID-19 vaccines were introduced. The primary objective of this study was to investigate the willingness of Chinese medical students to vaccinate against COVID-19 and the factors of their willingness to vaccinate.

## Methods

### Ethics Statement

This study was approved by the Wannan Medical College Ethics Committee.

### Survey Design and Participants

We conducted a web-based cross-sectional survey using an online questionnaire between 1 March and 12 March 2021. The survey was conducted by the Sojump (https://www.wjx.cn/) platform, considering its easy accessibility to the student participants. The participants in this study were students from a local medical school in Anhui, China. Eligible students were students enrolled in any grade of the undergraduate health care profession program majoring in a specialty area (clinical medicine, pharmaceutics, radiology, anesthesiology, psychology, nursing, stomatology, preventive medicine). The researchers shared electronic questionnaires *via* social media (such as QQ groups, WeChat App). The questionnaire online link was sent to participants through various social media, such as WeChat, QQ, etc. These participants were then encouraged to forward the WeChat groups of college students. Finally, 3,100 students from this school accessed the survey link, 3,047 of whom completed it correctly.

### Instruments

At the beginning of the survey, informed consent for participation was obtained from all participants. Participants could withdraw from the survey at any time. The survey was conducted anonymously, and information confidentiality was assured. A preliminary pilot experiment was conducted on 30 health care students interned in Yijishan Hospital, the Cronbach's alpha coefficients of the questionnaire internal consistency reliability as 0.87, indicating that the questionnaire can accurately measure the degree of vaccination behavior of health care students. The final questionnaire included the following parts:

#### Demographic

This section collects information about the general characteristics of the survey respondents, including gender, age, major, school year, place of residence, whether relatives and friends are medical staff, self-rated health, concerns about the COVID-19 vaccine, etc.

#### Attitude Toward COVID-19 Vaccine

We developed a brief, six-item scale based on prior studies of COVID-19 vaccine (8, 9). Each item was answered along a 5-point continuum ranging from Strongly disagree to Couldn't agree more. There are six questions about the anti-COVID-19 vaccine related attitudes. Participants' attitude toward COVID-19 vaccine was categorized, using Bloom's cut-off point. A score of ≥80% (≥24 points) is considered as positive attitude, and a score of <80% (<24 points) is considered as negative attitude (10).

#### EHealth Literacy Scale

The eHealth Literacy Scale (eHEALS) was compiled in 2006 by Norman and Skinner (11). Guo et al. Chineseized and revised the scale in 2013 (12). There are a total of eight items, including application, evaluation, and decision-making. The Likert 5-level scoring method is used. From “very inconsistent” to “very consistent”, 1–5 points are counted, and the total score is 8–40 points. A score of ≥32 is considered as qualified for electronic health literacy, and a score of <32 is considered as unqualified for electronic health (13). Cronbach's alpha coefficient of eHEALS was 0.975.

#### Statistical Analysis

All data analysis was performed using SPSS version 26.0 (IBM Corp., Armonk, NY, USA), frequencies and proportions were used to describe the demographic characteristics and attitudes toward COVID-19 vaccination. The chi-square test and Fisher's exact test was used to verify the differences between/in categorical variables (i.e., demographics, attitudes, eHealth literacy, sources of information, Whether relatives and friends are medical staff) related to each of the main outcomes (willingness to be vaccinated yes/no), with two-tailed *p*-value < 0.05 was considered statistically significant. Next, binary logistic regression analysis was used to examine the independent factors related to COVID-19 vaccine acceptance. The dependent variable was the willingness to be vaccinated (no = 0, yes = 1), with the significant factors in univariate analyses included as independent variables. The test level was a = 0.05, that is, a *p*-value of < 0.05 was considered statistically significant. Odds ratios (ORs) and 95% confidence intervals (CIs) were used to estimate associations.

#### Ethical Consideration

This study was conducted following the principles of the ethical guidelines of the Declaration of Helsinki 1995 (revised in 2013). An electronic informed consent form was provided at the beginning of the online questionnaire, and participants were asked to sign the informed consent form before starting the survey.

## Results

### Demographic Characteristics

We collected a total of 3,047 questionnaires with a response rate of 98.29% (3,047/3,100). Among the sample of 3,047 medical student, 984 (32.3%) were male and 2,063 (67.7%) were female. 1,926 (63.2%), 694 (22.8%), and 427 (14.0%) of participants lived in a rural, Town, and City areas, respectively. Furthermore, the 1st, 2nd, 3rd, and 4th year of the participants were 1,446 (47.5%), 723 (23.7%), 538 (17.7%), and 340 (11.2%), respectively. The average age of the medical student was 22.0 years (SD 2.51 years, range 17–23 years). The demographic characteristics of the study participants are presented in Table 1.

**Table 1:** Sociodemographic characteristics of the study sample (*N* = 3,047).

**Variable**		***n***	**(%)**
Gender	Male	984	32.3
	Female	2,063	67.7
Place of residence	Rural	1,926	63.2
	Town	694	22.8
	City	427	14
School year	1st year	1,446	47.5
	2nd year	723	23.7
	3rd year	538	17.7
	4th year	340	11.2
The impact of COVID19 on you	Less	1,801	59.1
	General	976	32
	Greater	270	8.9
Whether relatives and friends are medical staff	No	1,728	56.7
	Yes	1,319	43.3
Self-rated health	Worse	67	2.2
	General	1,058	34.7
	Better	1,922	63.1
Concerns about the COVID-19 vaccine	Less	429	14.1
	General	2,213	72.6
	Greater	405	13.3

### Acceptability of COVID-19 Vaccine

The participants were asked about their willingness to be vaccinated, that is: if the COVID-19 vaccine is available, will you get it?” with answers “yes” or “no”, who responded “yes” were deemed willing to be vaccinated. Overall, 37.9% (1,154) of participants indicated that they would be vaccinated against COVID-19, while 62.1% (1,893) declared that they would not. When comparing the willingness to vaccinate against COVID-19 or not, a significant difference was found in 5 items, such as, attitudes to the COVID-19 vaccine, levels of eHealth literacy, the impact of COVID19 on you, concerns about the COVID-19 vaccine and gender (Table 2).

**Table 2:** Analysis of variables for possible association with willingness to get COVID-19 vaccines.

**Variables**	**“COVID-19 vaccine is available, will you get it”**		**χ^2^**	***P*–value**
	**No (*n* = 1,893)**	**Yes (*n* = 1,154)**		
**Attitudes to the COVID-19 vaccine**			260.957	0.000
Negative	1,218 (75.5)	395 (24.5)		
Positive	675 (47.1)	759 (52.9)		
**Levels of eHealth literacy**			55.064	0.000
Low	1,113 (68.2)	519 (31.8)		
High	780 (55.1)	635 (44.9)		
**The impact of COVID19 on you**			27.539	0.000
Less	1,182 (65.6)	619 (34.4)		
General	572 (58.6)	404 (41.4)		
Greater	139 (51.5)	131 (48.5)		
**Place of residence**			3.115	0.211
Rural	1,175 (61.0)	751 (39.0)		
Town	440 (63.4)	254 (36.6)		
City	278 (65.1)	149 (34.9)		
**Whether relatives and friends are medical staff**			0.621	0.431
No	1,084 (62.7)	644 (37.3)		
Yes	809 (61.3)	510 (38.7)		
**Concerns about the COVID-19 vaccine**			72.597	0.000
Less	324 (75.5)	105 (24.5)		
General	1,379 (62.3)	834 (37.7)		
Greater	190 (46.9)	215 (53.1)		
**Gender**			22.453	0.000
Female	1,341 (65.0)	722 (35.0)		
Male	552 (56.1)	432 (43.9)		

### Logistic Regression for Analysis of the Factors Associated With the Willingness to Receive COVID-19 Vaccination

Gender (OR =1.222, 95% CI 1.036–1.441), the impact of COVID19 on you (OR =2.111, 95% CI 1.539–2.895), concerns about the COVID-19 vaccine (OR =2.111, 95% CI 1.539–2.895), attitude toward COVID-19 vaccines (OR =3.016, 95% CI 2.564–3.548) and eHealth literacy (OR =1.182, 95% CI 1.005–1.391) were also significant correlates of the willingness to vaccinate (Table 3).

**Table 3:** Binary logistic analysis of factors influencing willingness to receive the COVID-19 vaccination (*n* = 3,047).

**Variables**	**β**	**S.E**.	**Wald**	***P***	**OR**	**OR 95% CI**
**Gender**						
Female					1	
Male	0.201	0.084	5.678	0.017	1.222	1.036–1.441
**The impact of COVID19 on you**						
Less					1	
General	0.28	0.086	10.538	0.001	1.443	1.124–1.851
Greater	0.422	0.14	9.1	0.003	2.111	1.539–2.895
**Concerns about the COVID-19 vaccine**						
Less					1	
General	0.366	0.127	8.305	0.004	1.443	1.124–1.851
Greater	0.747	0.161	21.5	0.000	2.111	1.539–2.895
**eHealth literacy**						
No					1	
Yes	0.167	0.083	4.063	0.04	1.182	1.005–1.391
**Attitudes to the COVID-19 vaccine**						
Negative					1	
Positive	1.104	0.083	177.607	0.000	3.016	2.564–3.548

## Discussion

As far as we know, this was the first study that examined the attitudes of Chinese medical students toward the COVID-19 vaccine and their related factors. In this study, we found that 37.9% (1,154) of the Chinese medical students indicated that they would be vaccinated against COVID-19, lower than previous report (14). This finding is quite enlightening, as it is often believed that the attitudes of medical students toward vaccination will be positive due to their professional knowledge and training. However, this was not the case in this study. The main reasons for the low acceptance rate of vaccines in medical students may include the uncertainties about vaccine safety and efficacy, and insufficient knowledge about the potential benefits of vaccination. Study has found that the public's perception of the risks and benefits of vaccination constitutes the main obstacle to vaccine acceptance (15). Medical students, as part of the general population, are also vulnerable to subjective judgments that affect their behaviors and vaccination decisions, even though they have medical knowledge.

### Differences in Willingness to Receive the COVID-19 Vaccination

#### Gender

We found that COVID-19 Vaccination intentions was strongly associated with gender, men are more receptive to COVID-19 vaccines, which is in concordance with previously studies (16–18). Previous studies have shown that women are more cautious in accepting innovative technologies. Women may be worried about the impact of the vaccine on fertility and some women are allergic to vaccines (19). Thus, the willingness to COVID-19 vaccinate is lowest among female medical students.

### Relationship Between EHealth Literacy and Willingness to Receive the COVID-19 Vaccination

Findings from this study indicated that eHealth literacy is associated with COVID-19 vaccination. Medical students with higher eHealth literacy might be inclined to Vaccination with COVID-19, which is consistent with previous studies (20). Good e-health literacy means that individuals haves more and better information about COVID-19 vaccines obtained on the Internet, and have better ability to health self-management and adjustment. In a previous study, we found that nursing students with higher eHealth literacy had healthier COVID-19 protective behaviors (21). Therefore, it is very important to take measures to improve medical students' eHealth literacy to promote COVID-19 vaccine.

### The Impact of COVID-19

In our study, the impact of COVID19 was a factors of vaccine acceptance. The medical students who reported greater impact of COVID19 were more willing to receive the COVID-19 vaccination. These students may have stronger motivation to protect themselves, and the COVID-19 vaccination is seen as a useful means for protection. The COVID-19 pandemic isolation and recommendations of social distancing have fundamentally changed our society functions. Research has shown that sudden changes in life (home and work dynamics) could increase the risk of changes in sleep, physical symptoms, avoidance, irritability, and isolation (22). People who had more affected by the epidemic of COVID-19 would pay more attention to the COVID-19 vaccine and were more willing to receive vaccines.

### Concerns About the COVID-19 Vaccine

The COVID-19 pandemic has had a fatal impact on the economy, health system, education system and infrastructure of many countries (23). And, the disease currently has no cure, and the prognosis is poor. Concerns about the COVID-19 vaccine was a useful strategy, which could increase the willingness to tack the COVID-19 vaccine (24). Those who are concerned about the COVID-19 vaccine would learn about the evidence-based information in various ways, which would improve the awareness of the vaccine itself, and reduce skepticism and promote acceptance.

### Attitudes to the COVID-19 Vaccine

Our findings indicate that it would be effective to increase positive attitude toward COVID-19 vaccination, which was found to be a facilitating factor. The positive attitude to the COVID-19 vaccine could increase the willingness to tack the COVID-19 vaccine (25). And students whose families had been vaccinated were able to view the COVID-19 vaccine with a more positive attitude (26). The most negative attitude toward the COVID-19 vaccines was the concern about the side effects of the vaccine. Factors such as perceptions, trust in vaccines, and trust in vaccine providers have been found to contribute to vaccine hesitancy and refusal (27, 28).

This study had some limitations. First, the results of this study come from one medical college, and the extrapolation of the results is not necessarily applicable to non-medical students. Second, participants were selected purposively using snowball sampling, so there could be some deficiencies in the quality control of the questionnaire, which will affect the research results. And, this study was conducted 3 months after the release of the vaccine, which only reflect the initial stage of the vaccination willingness. Further research is still needed to analyze the middle and later stages of willingness to receive the COVID-19 vaccination and the actual vaccination behavior.

## Conclusions

In conclusion, the present study revealed that the willingness to receive COVID-19 vaccination was sub-optimal among medical students in China. And those who don't want to be vaccinated need more attentions. The better the level of attitude and the higher the eHealth literacy, the more positive the willingness of medical students to vaccinate. The safety and side effects of the vaccine remained the major factors of reluctance to vaccinate. Because of the important role in the fight against the COVID-19 pandemic and other infectious diseases, it is critical to increase the acceptance of vaccines among medical students. In addition, clear policy related to the COVID-19 vaccine needs are needed to deal with negative views of the vaccine.

## Data Availability Statement

The raw data supporting the conclusions of this article will be made available by the authors, without undue reservation.

## Ethics Statement

The studies involving human participants were reviewed and approved by the Ethics Committee of Nursing at Wannan Medical College (Wuhu, China). The patients/participants provided their written informed consent to participate in this study.

## Informed Consent Statement

Participants have been informed before answering the questions. They were informed that participation in the survey was voluntary and anonymous. Participation can be canceled at any time. In addition, they were told that the results of the questions would be evaluated and published.

## Author Contributions

HL and MZ: conceptualization and writing—original draft preparation. HL: methodology. ZZ: software. XT, HL, and MZ: validation. LH: formal analysis. EZ: investigation. LY: resources. SD: data curation. All authors have read and agreed to the published version of the manuscript.

## Funding

This research was funded by MOE (Ministry of Education in China) Project of Humanities and Social Sciences (20YJC190006), the Teaching Quality and Teaching Reform Project of Wannan Medical College (2019jyxm47), the Teaching Quality and Teaching Reform Project of Anhui Provincial Department of Education (2020jyxm2076), the Teaching Quality and Teaching Reform Project of Wannan Medical College (2020jyxm58), and the Key Research Base of Humanities and Social Sciences in Anhui Province: Project of the Research Center for Mental Health Education of College Students (SJD202110).

## Conflict of Interest

The authors declare that the research was conducted in the absence of any commercial or financial relationships that could be construed as a potential conflict of interest.

## Publisher's Note

All claims expressed in this article are solely those of the authors and do not necessarily represent those of their affiliated organizations, or those of the publisher, the editors and the reviewers. Any product that may be evaluated in this article, or claim that may be made by its manufacturer, is not guaranteed or endorsed by the publisher.
